# Amelioration of hyperglycaemia and modulation of antioxidant status by *Alcea rosea* seeds in alloxan-induced diabetic rats

**DOI:** 10.1080/13880209.2017.1333127

**Published:** 2017-06-01

**Authors:** Parvaiz A. Dar, Fasil Ali, Ishfaq A. Sheikh, Showkat A. Ganie, Tanveer A. Dar

**Affiliations:** a Clinical Biochemistry, University of Kashmir, Srinagar, India;; b King Fahd Medical Research Center, King Abdulaziz University, Jeddah, Kingdom of Saudi Arabia

**Keywords:** Hyperglycaemic, oxidative stress, glutathione, antidiabetic, phenolic compounds

## Abstract

**Context:***Alcea rosea* L. (Malvaceae) has various medicinal uses including anticancer, anti-inflammatory and analgesic properties. However, there is no report on its antidiabetic activity.

**Objective:***Alcea rosea* seed extracts were evaluated for antihyperglycaemic and antioxidative potential in diabetic rats.

**Materials and methods:** Single intra-peritoneal injection of alloxan (130 mg/kg b.w.) was used for induction of diabetes in Albino Wistar rats. Antihyperglycaemic and antioxidant activities of methanol and aqueous extracts of *Alcea rosea* seed (100 and 300 mg/kg b.w.), administered orally on daily basis for 15 days, were assessed *in vivo* for fasting blood glucose level and antioxidant status of liver and pancreas. Metformin was used as a positive control.

**Results:** Aqueous and methanol extracts (300 mg/kg b.w.) decreased blood glucose level in diabetic rats by 24% and 46%, respectively. Administration of aqueous and methanol extracts at 300 mg/kg b.w. significantly (*p* < 0.01) modulated the antioxidant status of liver in diabetic rats by increasing levels of GR (22.5 ± 1.0, 24.4 ± 1.02 μg GSSG utilized/min/mg of protein), GPx (20.7 ± 1.2, 23.6 ± 2.04 μg GSH utilized/min/mg of protein), SOD (36.1 ± 1.7, 39.05 ± 1.5 units/mg of protein) and CAT (1744.5 ± 132.5, 1956.6 ± 125.2 nmol H_2_O_2_ decomposed/min/mg of protein), respectively. Similar results were observed for pancreas.

**Discussion and conclusions:** Antihyperglycaemic and antioxidative potentials of *Alcea rosea* seeds suggest its usefulness in management of diabetes and its complications. This is the first report on antidiabetic activity of this plant.

## Introduction

Diabetes mellitus, a metabolic syndrome, is primarily characterized by hyperglycaemia caused due to the abnormal insulin secretion, insulin action or both (Aggarwal et al. 2014; American Diabetes Association 2014). Recent studies have predicted that prevalence of diabetes, at global level, will double from 170 million in 2000 to 360 million in 2030 with highest increase in India (Wild et al. 2004). In fact, it is estimated that diabetes may afflict up to 79 million people in India by 2030 (Whiting et al. 2011). Despite the availability of a large number of known antidiabetic drugs in the market, diabetes mellitus and its associated complications continue to be a major medical problem (Eurich et al. 2007). The major reason for this is that currently used antidiabetic drugs, besides being costly, are associated with severe side effects such as development of hypoglycaemia, liver toxicity, weight gain and gastrointestinal disturbances (Davidson 1991; Rodbard et al. 2007). However, herbal medicines have been perceived to be safe and effective, with minimal side effects, and are relatively cheap (Jouad et al. 2001; Modak et al. 2007; Malviya et al. 2010; Pareek et al. 2009). In view of the adverse effects associated with conventional antidiabetic drugs and also the safety, cheapness and effectiveness of herbal medicines, traditionally used medicinal plants may be explored for their antidiabetic activity (Li et al. 2004).

Keeping the above in consideration and the hunt for new potent antidiabetic agents, this study was carried out to explore the antihyperglycaemic effect of one of the widely grown medicinal plant of Kashmir Himalayas, *Alcea rosea* L. (Malvacea). This plant is associated with various biological activities such as anticancer (Ahmed et al. 2016), antiurolithiatic, diuretic anti-inflammatory (Ahmadi et al. 2012), antibacterial (Lim 2014), hepatoprotective (Hussain et al. 2014), analgesic and cytotoxic activities (Wang et al. 1989). Interestingly so far no antidiabetic study has been carried out on this plant species. *A. rosea*, commonly known as hollyhock, is an ornamental plant widely grown in temperate regions and various tropical hilly areas throughout the world. Morphologically, *A. rosea* is a slender, erect, sparsely branched herbaceous biennial or short-lived perennial plant.

## Materials and methods

### Chemicals

Alloxan monohydrate was purchased from CDH, Pvt. Ltd., New Delhi, India. Metformin tablets (Franco-Indian Pharmaceuticals Pvt. Ltd., Mumbai, India) were purchased from the authorized distributor of the company. All other chemicals used for experimental studies were of analytical grade.

### Plant material

The seeds of *Alcea rosea* were collected from Vanaspati Medicinal Plant Nursery Ganderbal in the months of September–October 2013, and were authenticated by Dr. Akhter Hussain Malik, Curator ‘Centre for Plant Taxonomy’, Department of Botany, University of Kashmir. The authenticated plant seed material was dried in shade at room temperature. A reference specimen has been deposited at the Herbarium of Department of Botany, University of Kashmir under voucher specimen number KASH-bot/KU/CS-735-PAD.

### Preparation of extract

The shade-dried seed material was coarsely powdered by using mortar and pestle and filtered by a sieve of size 0.3 mm. The powdered seed material was successively extracted with methanol and distilled water by using Soxhlet extractor. Extract obtained was then concentrated by using rotary evaporator under reduced pressure. One hundred grams of *Alcea rosea* seed powder successively yields 9.7 g (9.7%) and 7.4 g (7.4%) methanol and aqueous extract, respectively. Solid extract obtained was stored at –20 °C for further use.

### Experimental animals

Adult male Albino rats of Wistar strain weighing 200–250 g, obtained from animal house, University of Kashmir, were used throughout this study. The animals were provided with water and pellet diet procured from Hindustan Lever, Ltd., Mumbai, India. Animals were housed in well maintained cages under standard husbandry conditions (12 h light/dark cycle; 25 ± 0.5 °C). Animals described as fasting were deprived of food and water for 16 h. Animals used in the present study were maintained in accordance with the guidelines prescribed by the National Institute of Nutrition, Indian Council of Medical Research, India and were duly approved by Institutional Ethical Committee.

### Toxicity study

In order to determine toxicity, different doses of the extracts (50–500 mg/kg b.w.) were orally administered to different groups of rats (six rats in each group). Mortality as well as behavioural changes of these tested animals was observed periodically for next 48 h and once daily up to a week after the administration of extracts.

### Induction of experimental diabetes in rats

Prior to induction of diabetes, animals were put on fast for 24 h. Single intra-peritoneal injection of freshly prepared alloxan (130 mg/kg b.w.) in normal saline was used for induction of experimental diabetes (Negres et al. 2003; Mule et al. 2016). After 72 h, animals with fasting blood glucose levels of >200 mg/dL were considered diabetic and selected for further experimentation.

### Experimental protocol

A total of 42 albino Wistar rats were used and divided into seven groups of six rats each.

Group I: Served as normal control and received distilled water only (1 mL/day).

Group II: Served as negative control (diabetic) and received only distilled water (1 mL/day).

Group III: Diabetic animals were treated with standard drug metformin (100 mg/kg b.w./day).

Group IV: Diabetic animals were treated with aqueous extract of *Alcea rosea* seed (ARA 100 mg/kg b.w./day).

Group V: Diabetic animals were treated with aqueous extract of *Alcea rosea* seed (ARA 300 mg/kg b.w./day).

Group VI: Diabetic animals were treated with methanol extract of *Alcea rosea* seed (ARM 100 mg/kg b.w./day).

Group VII: Diabetic animals were treated with methanol extract of *Alcea rosea* seed (ARM 300 mg/kg b.w./day).

### Measurement of blood glucose level

In experimental diabetic rats, blood glucose levels were measured at the start and after weakly intervals. Accusure glucometre (Make-Bionime Corporation, Taichung City, Taiwan) was used for measurement of blood glucose level. Blood samples from experimental rats were obtained by tail vein puncture.

### Determination of total phenolic content

Spectrophotometric method of Singleton et al. (1999) was used for estimation of phenolic content in plant extracts. Aqueous and methanol extracts were prepared with a concentration of 1 mg/mL. The reaction mixture consisted of 0.5 mL of extract solution, 2.5 mL of 7.5% NaHCO_3_ and 2.5 mL of 10% Folin–Ciocalteu’s reagent. The reaction mixture was incubated for 45 min at 45 °C. Absorbance of the reaction mixture was measured at 765 nm against blank. Quantification of phenolic compounds, expressed in terms of gallic acid equivalent (mg of GA/g of extract), was evaluated on the basis of standard curve of gallic acid.

### Estimation of lipid peroxidation

Lipid peroxidation in liver and pancreas was estimated colorimetrically, using malondialdehyde (MDA) as an oxidative stress marker, by the method of Niehaus and Samuelson (1968). In brief, 0.1 mL of tissue homogenate was treated with 2 mL of (1:1:1 ratio) TCA–TBA–HCl reagent (15% trichloro acetic acid (TCA):0.37% thiobarbituric acid (TBA):0.25 N HCl) and was placed in water bath for 15 min. After cooling, the homogenate was centrifuged and absorbance of the clear supernatant was measured at 535 nm against a reference blank.

### Estimation of glutathione level

The spectrophotometric assay procedure of Moron et al. (1979) was followed for estimation of GSH level. Actually the acid soluble sulphydryl groups (glutathione (GSH)) on reaction with DTNB (5,5′-dithiobis-2-nitrobenzoic acid) forms a yellow coloured complex with a maximum absorbance at 412 nm (Moron et al. 1979). For this, 100 μL of 25% TCA was used to precipitate the 500 μL tissue homogenate and was centrifuged at 3000 rpm for 10 min. After centrifugation, 100 μL of supernatant was added to the reaction mixture containing 0.9 mL of 0.2 mM sodium phosphate buffer pH 7.4 and 2 mL of 0.6 mM DTNB. Formation of yellow coloured complex was measured against the blank at 412 nm. Glutathione content was estimated using DTNB with a molar extinction coefficient of 13,100 M^−1^ cm^−1^.

### Glutathione peroxidase

Glutathione peroxidase (GPx) activity was evaluated using the method of Sharma et al. (2000). The reaction mixture consisted of 1.49 mL of sodium phosphate buffer (0.1 M, pH 7.4), 0.1 mL EDTA (1 mM), 0.1 mL sodium azide (1 mM), 0.1 mL of 1 mM GSH, 0.1 mL of NADPH (0.02 mM), 0.01 mL of 1 mM H_2_O_2_ and 0.1 mL serum in a total volume of 2 mL. NADPH oxidation was measured spectrophotometrically at 340 nm and the activity of enzyme was calculated as nmoles of NADPH oxidized/min/mg of protein, using molar extinction coefficient of 6.22 × 10^3^ M^−1^ cm^−1^.

### Glutathione reductase activity

Glutathione reductase (GR) activity was evaluated by the spectrophotometric method of Sharma et al. (2000). The reaction mixture contained 1.6 mL of sodium phosphate buffer (0.1 M, pH 7.4), 0.1 mL EDTA (1 mM), 0.1 mL of 1 mM oxidized glutathione, 0.1 mL of NADPH (0.02 mM), 0.01 mL of 1 mM H_2_O_2_ and 0.1 mL tissue homogenate in a total volume of 2 mL. Activity of GR measured against the blank at 340 nm was expressed as nmoles of NADPH oxidized/min/mg of protein using molar extinction coefficient of 6.22 × 10^3^ M^−1^ cm^−1^.

### Glutathione-S-transferase (GST) activity

For quantification of GST activity, spectrophotometric method of Haque et al. (2003) was used. The reaction assay consisted of 0.1 mL tissue homogenate, 1.67 mL of 0.1 M sodium phosphate buffer pH 6.5, 0.2 mL of 1 mM GSH and 0.025 mL of 1 mM CDNB (1-chloro-2,4-dinitrobenzene). Change in absorbance was spectrophotometrically measured at 340 nm and activity of GST was expressed as nmoles of CDNB conjugates formed/min/mg protein using molar extinction coefficient of 9.6 × 10^3^ M**^−^**^1^ cm**^−^**^1^.

### Catalase activity

Activity of catalase enzyme in tissue homogenate was estimated by the method of Claiborne (1985). The reaction mixture consisted of 0.05 mL tissue homogenate, 1.95 mL of 0.05 M phosphate buffer pH 7.0 and 1.0 mL of 0.019 M H_2_O_2_. Absorbance change was spectrophotometrically measured at 240 nm and the activity of catalase was expressed in terms of nmoles of H_2_O_2_ consumed/min/mg of protein.

### Superoxide dismutase activity

Superoxide dismutase (SOD) activity was quantified by the method of Beauchamp and Fridovich (1971). The SOD activity was determined in a reaction mixture consisting of 0.5 mL of tissue homogenate, 0.2 mL of 0.1 mM EDTA, 1 mL of 50 mM sodium carbonate and 0.4 mL of 25 μM NBT (nitro blue tetrazolium). For initiation of reaction, 0.4 mL of 1 mM hydroxylamine–hydrochloride was added to the reaction mixture. Absorbance change was spectrophotometrically measured at 560 nm. Activity of SOD was expressed as the amount of SOD that inhibits 50% reduction of NBT.

### Statistical analysis

The data were presented as mean ± SD and evaluated by one-way ANOVA followed by the Bonferroni *t*-test to detect inter group differences. Differences were considered to be statistically significant if *p* < 0.05.

## Results

### Total phenolic content

**Figure 1.**
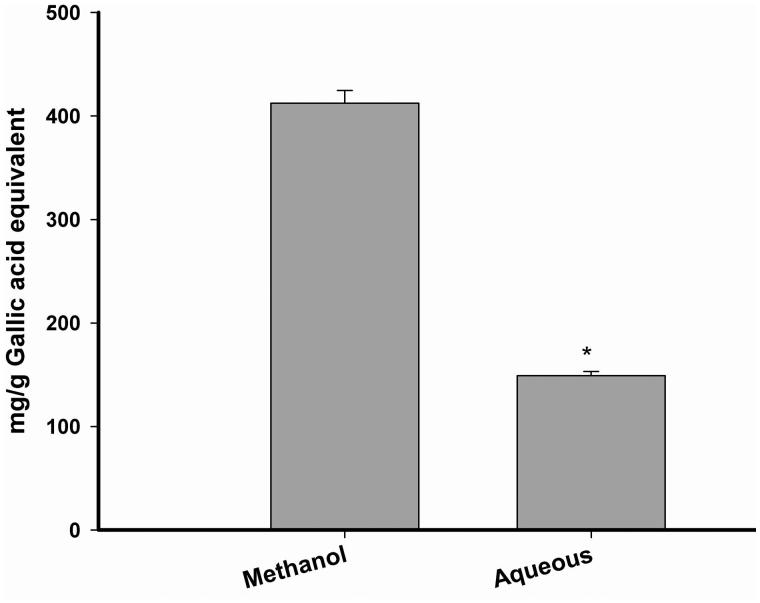
Total phenolic content of Alcea rosea seed extracts. The experiments were carried out in triplicates.

Both aqueous and methanol extract were found to possess significant amount of phenolic content (Figure 1). However, methanol extract was found to possess higher content of phenolic components (412.23 mg/g gallic acid equivalent) as compared to the aqueous one (149.01 mg/g gallic acid equivalent).

### Toxicity study

Both aqueous and methanol extracts of *Alcea rosea* seeds showed nontoxic effects on the observed behavioural parameters of normal healthy rats. A survival rate of 100% was observed in animals treated with 50–300 mg/kg b.w. of *A. rosea* extracts while as from 350 to 500 mg/kg b.w. of the extracts studied, a survival rate of 90–50% was observed.

### Antihyperglycaemic activity of *A. rosea* extracts

Antihyperglycaemic activity of methanol and aqueous extracts of *Alcea rosea* seeds was evaluated by measuring fasting blood glucose level at weekly intervals until the end of study. Results presented in Table 1 show that fasting blood glucose level in diabetic rats was found to be 3–4 times higher than that of normal ones with a consistent increase in the blood glucose levels of diabetic rats (group II) throughout the study. A good decline in blood glucose level was observed at both concentrations of the extracts used. However, methanol extract at 300 mg/kg b.w. exhibited a significant (*p* < 0.001) (46.2%) antihyperglycaemic effect, quite comparable to that of the known metformin drug (66.8%).

**Table 1.** Variation in fasting blood glucose level after oral administration of *Alcea rosea* extracts in alloxan induced diabetic rats.

Fasting blood glucose level (mg/dl)			
Groups	0th day	7th day	15th day
Group I (Normal)	100.5 ± 9.8	92.7 ± 9.3	101 ± 4.6
Group II (Diabetic)	360.7 ± 15.4^$^	377.5 ± 13.1^$^	408.5 ± 10.5^$^
Group III (Metformin)	352.3 ± 10.05^$^^,^^#^	201.09 ± 9.6^$^^,^^#^	116.8 ± 11.53^$^^,^^#^
Group IV (ARA 100)	370.2 ± 8.2^$^^,^^#^^,^*	351.2 ± 8.4^$^^,^^#^^,^*	306.7 ± 11.9^$^^,^^#^^,^*
Group V (ARA 300)	361.5 ± 10.7^$^^,^^Δ^^,^*	310.2 ± 10.6^$^^,^^#^^,^*	274.7 ± 14.6^$^^,^^#^^,^*
Group VI (ARM 100)	365.7 ± 11.1^$^^,^^Δ^^,^*	289.5 ± 5.9^$^^,^^#^^,^*	259.5 ± 6.60^$^^,^^#^^,^*
Group VII (ARM 300)	370.2 ± 12.7^$^^,^^#^^,^*	268.5 ± 6.7^$^^,^^#^^,^*	199.2 ± 7.1^$^^,^^#^^,^*

The values were presented as mean ± SD for six animals in each observation and evaluated by one way ANOVA followed by the Bonferroni *t*‐test to detect inter group differences.

$ *p*< 0.001, as compared with normal control group.

# *p*< 0.001, as compared with Diabetic group.

* *p*< 0.001, as compared with Metformin.

Δ Nonsignificant as compared with Metformin.

### Effect of extracts on lipid peroxidation

**Figure 2.**
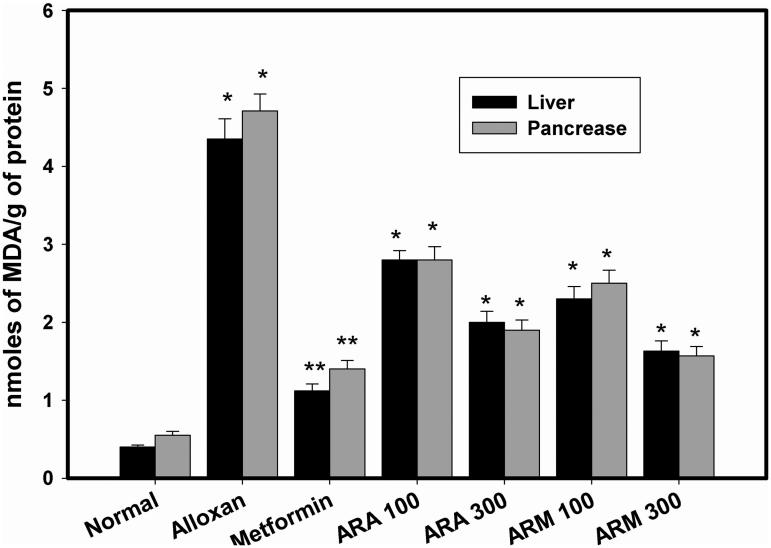
Effect of aqueous and methanol extracts of *Alcea rosea* seed on lipid peroxidation of liver and pancreas in alloxan induced diabetic rat models. *p < 0.001, as compared with normal control group. **p < 0.001 as compared with diabetic group. Each value is a mean ± SD (*n* = 6 in each group). The experiments were carried out in triplicates.

As compared to normal group (Figure 2), alloxan induced diabetes (Alloxan group) caused significant (*p* < 0.01) increase in MDA level from 0.40 ± 0.025 to 4.35 ± 0.26 nmol of MDA formed/mg protein and 0.55 ± 0.05 to 4.715 ± 0.22 nmol of MDA formed/mg protein in liver and pancreatic tissue, respectively. Treatment of diabetic rats with different doses of aqueous and methanol extracts exhibited antilipid peroxidation effect by reducing MDA level towards normal level in both the organs (Figure 2). Methanol extract (300 mg/kg b.w.) showed highest antilipid peroxidation activity (1.63 ± 0.13 and 1.57 ± 0.12 nmol of MDA formed/g of protein in liver and pancreas, respectively), which is comparable to that of metformin drug (1.12 ± 0.09 and 1.40 ± 0.11 nmol of MDA formed/g of protein in liver and pancreas, respectively).

## Effect of extracts on antioxidant status of diabetic rat models

### Glutathione level in liver and pancreas

**Figure 3.**
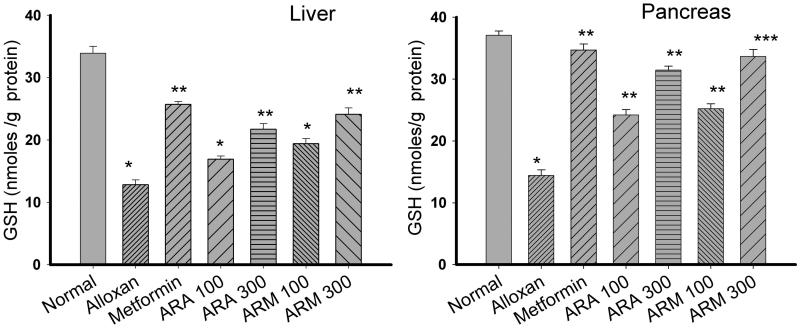
Effect of aqueous and methanol extracts of *Alcea rosea* seed on glutathione level of liver and pancreas in alloxan induced diabetic rat models. *p < 0.001, as compared with normal control group. **p < 0.001 as compared with diabetic group. ***p < 0.001 as compared with metformin group. Each value is a mean ± SD (*n* = 6 in each group). The experiments were carried out in triplicates.

As compared to normal rats (33.9 ± 2.4 and 37.07 ± 0.71 nmol/g of protein, glutathione level in liver and pancreas, respectively), a significant (*p* < 0.01) decrease in glutathione level in liver and pancreas was observed in the untreated alloxan group II (12.8 ± 1.7 and 14.41 ± 0.91 nmol/g of protein, respectively) of diabetic rats (Figure 3). Oral administration of aqueous and methanol extracts of *Alcea rosea* seeds for 15 consecutive days showed a significant (*p* < 0.01) concentration dependent increase in the GSH status. Methanol extract at a concentration of 300 mg/kg b.w. showed comparable protective effect on both the tissues (24.1 ± 1.1 and 33.65 ± 1.15 nmol/g of protein in liver and pancreas, respectively) with that of known standard metformin drug (25.7 ± 1.7 and 34.68 ± 0.99 nmol/g of protein in liver and pancreas, respectively).

### Glutathione reductase level

Glutathione reductase content in liver and pancreas of diabetic rats (group II of Tables 2 and 3) was observed to (12.8 ± 1.7 and 14.41 ± 0.91 μg GSSG utilized/min/mg of protein, respectively), exhibited significant diminution (*p* < 0.01) as compared to normal rats which was observed to be 33.9 ± 2.4 and 37.07 ± 0.7 μg GSSG utilized/min/mg of protein, in liver and pancreas, respectively (group I of Tables 2 and 3). However, treatment with different concentrations of aqueous and methanol extracts of *Alcea rosea* seed restored the GR levels in both the tissues, in a dose dependent manner. As expected, methanol extract at 300 mg/kg b.w. significantly (*p* < 0.01) restored the GR level towards normal in both liver (24.4 ± 1.02 μg GSSG utilized/min/mg of protein) and pancreatic (13.74 ± 0.88 μg GSSG utilized/min/mg of protein) tissue of diabetic rats (group VII in Tables 2 and 3), which was quite comparable to that of the metformin drug (27.1 ± 2.1 and 12.78 ± 0.94 μg GSSG utilized/min/mg of protein, in liver and pancreatic tissue of group III rats, respectively) used in this study.

**Table 2.** Effect of aqueous and methanol extracts of *Alcea rosea* seed on enzymatic and nonenzymatic antioxidant status of liver in alloxan induced diabetic rats.

Enzymes	Group I	Group II	Group III	Group IV	Group V	Group VI	Group VII
GR (μg GSSG utilized/min/mg of protein)	35.9 ± 7.3	10.5 ± 2.3*	27.1 ± 2.1*^,^^#^	15.5 ± 1.8*^,^^#^	22.5 ± 1.0*^,^^#^	18.4 ± 1.3*^,^^#^	24.4 ± 1.02*^,^^#^
GPx (μg GSH utilized/min/mg of protein)	38.1 ± 4.1	6.2 ± 2.1*	25.5 ± 2.01*^,^^#^	11.9 ± 2.1*^,^^#^	20.7 ± 1.2*^,^^#^	13.3 ± 1.2*^,^^#^	23.6 ± 2.04*^,^^#^
GST (nmol of CDNB conjugated/min/mg of protein)	32.02 ± 3.5	17.9 ± 1.4*	25.8 ± 1.02*^,^^#^	19.06 ± 0.7*^,^^$^	22.7 ± 1.8*^,^^#^	20.66 ± 1.1*^,^^#^	23.6 ± 2.30*^,^^#^
SOD (units/mg of protein)	48.3 ± 4.01	25.05 ± 3.2*	42.01 ± 2.08*^,^^#^	30.7 ± 2.6*^,^^#^	36.1 ± 1.7*^,^^#^	31.4 ± 1.4*^,^^#^	39.05 ± 1.5*^,^^#^
CAT (nmol H_2_O_2_ decomposed/min/mg of protein)	2630.8 ± 187.5	443.6 ± 62.9*	2134.8 ± 167.9*^,^^#^	854.1 ± 119.7*^,^^#^	1744.5 ± 132.5*^,^^#^	1131.8 ± 84.04*^,^^#^	1956.6 ± 125.2*^,^^#^

The data are presented as mean ± SD and evaluated by one-way ANNOVA followed by the Bonferroni *t*-test to detect intergroup differences.

* *p* < 0.01 as compared with normal control.

# *p* < 0.01 as compared to alloxan treated group.

$ Non-significant as compared to alloxan group.

**Table 3.** Effect of aqueous and methanol extracts of *Alcea rosea* seed on enzymatic and nonenzymatic antioxidant status of pancreas in alloxan induced diabetic rats.

Enzymes	Group I	Group II	Group III	Group IV	Group V	Group VI	Group VII
GR (μg GSSG utilized/min/mg of protein)	14.53 ± 2.2	6.47 ± 1.26*	12.78 ± 0.94^a^^,^^#^	9.18 ± 0.85*^,^^#^	11.99 ± 0.85*^,^^#^	9.90 ± 0.41*^,^^#^	13.74 ± 0.88^a^^,^^#^
GPX (μg GSH utilized/min/mg of protein)	6.13 ± 0.9	3.39 ± 0.48*	5.58 ± 0.46^a^^,^^#^	3.53 ± 0.61*^,^^#^	4.57 ± 0.35^b^^,^*	3.79 ± 0.33^b^^,^*	5.33 ± 0.35^a^^,^*
GST (nmol of CDNB conjugated/min/mg of protein)	118.90 ± 1.5	60.88 ± 2.90*	107.70 ± 2.78*^,^^#^	78.01 ± 2.54^b^^,^*	96.47 ± 2.11*^,^^#^	82.15 ± 2.10*^,^^#^	103.1 ± 4.4*^,^^#^
SOD (units/mg protein)	54.05 ± 3.2	34.54 ± 3.26*	46.76 ± 1.83*^,^^#^	37.94 ± 1.40*^,^^#^	43.23 ± 2.99*^,^^#^	40.29 ± 1.29*^,^^#^	46.27 ± 0.83*^,^^#^
CAT (nmol H_2_O_2_ decomposed/min/mg of protein)	2206.1 ± 78.8	908.1 ± 104.4*	1779.3 ± 29.1*^,^^#^	1098.1 ± 10.3*^,^^#^	1578.9 ± 43.9*^,^^#^	1178.1 ± 86.1*^,^^#^	1689.1 ± 71.1*^,^^#^

The data are presented as mean ± SD and evaluated by one-way ANNOVA followed by the Bonferroni *t*-test to detect inter group differences.

* *p* < 0.01 as compared with normal group.

# *p* < 0.01 as compared to alloxan treated group.

a Non-significant as compared to normal group.

b Non-significant as compared to alloxan group.

### Glutathione peroxidase level

Glutathione peroxidase activity in untreated diabetic rats (group II) was found to decrease to a value of 6.2 ± 2.1 and 3.39 ± 0.48 μg GSH utilized/min/mg of protein in both liver (Table 2) and pancreas (Table 3), respectively. However, oral administration of *Alcea rosea* extracts in diabetic rats restored the GPx activity in both liver and pancreatic tissue significantly (*p* < 0.01). Methanol extract at 300 mg/kg b.w./day was found to have a comparable GPx restoration potential to that of the metformin drug (Tables 2 and 3).

### Glutathione S-transferase level

Results obtained in analysing the effect of two extracts of *Alcea rosea* on GST activity in liver and pancreatic tissue of experimental rat models are shown in Tables 2 and 3. GST level, as compared to normal group, was found to decrease in untreated diabetic rats (group II) with a value of 44.09% and 48.79% in liver and pancreatic tissue, respectively. However, aqueous and methanol extracts of *Alcea rosea* seed showed potent antioxidant activity by increasing GST content in treated groups. Among the two extracts, methanol extract at 300 mg/kg b.w./day (ARM 300) exhibited reasonable antioxidant effect by improving GST (24.15% and 39.79% in liver and pancreas of treated diabetic rats, respectively, *p* < 0.01) level which is comparable to that of the standard metformin drug alone (30.62% and 43.47% in liver and pancreas, respectively).

### Superoxide dismutase levels

In untreated diabetic group (group II), diabetes caused significant (*p* < 0.01) reduction of the SOD activity in both liver (Table 2) and pancreatic tissue (Table 3) to a value of 25.05 ± 3.2 and 34.54 ± 3.26 units/mg of protein, respectively. However, oral administration of aqueous and methanol extract caused significant (*p* < 0.01) improvement in SOD level in both tissues. In both liver and pancreatic tissue of methanol extract (300 mg/kg b.w.) treated groups (group VII in Tables 2 and 3), the SOD level was found to reach almost to same level (39.05 ± 1.5 and 46.27 ± 0.83 units/mg of protein in liver and pancreas, respectively, *p* < 0.01) as that of the standard metformin drug (42.01 ± 2.08 and 46.76 ± 1.83 units/mg of protein in liver and pancreas, respectively).

### Catalase levels

In untreated diabetic rats (group II), alloxan caused a significant (*p* < 0.01) reduction of catalase level to 443.6 ± 62.9 and 908.12 ± 104.40 nmol/min/mg of protein in liver (Table 2) and pancreas (Table 3) respectively as compared to that of normal group, (group I of Tables 1 and 2) with a value of 2630.8 ± 387.5 and 2206.13 ± 78.89 nmol/min/mg of protein in liver and pancreas, respectively. Oral administration of aqueous and methanol extracts for 15 consecutive days caused a significant (*p* < 0.01) improvement in catalase level in both liver and pancreatic tissue as compared to that of untreated diabetic animals (group II). The catalase restorative activity exhibited by the *A. rosea* seed extracts, as shown in Tables 2 and 3 is quite significant (*p* < 0.01). However, among the two extracts, methanol extract at 300 mg/kg b.w. shows comparable catalase restorative activity (1956.6 ± 125.2 and 1689.1 ± 71.1 nmol H_2_O_2_ decomposed/min/mg of protein in liver and pancreas, respectively) to that of standard metformin drug (2134.8 ± 167.9 and 1779.3 ± 29.1 nmol H_2_O_2_ decomposed/min/mg of protein in liver and pancreas, respectively).

## Discussion

Evaluation of antidiabetic and antioxidative potential of *Alcea rosea* seed extracts in alloxan-induced diabetic rats was achieved through determination of (i) their antidiabetic activity (antihyperglycaemic effect) and (ii) the ability of the extracts to stimulate cellular antioxidant defence system in diabetic rats. Antihyperglycaemic activity results clearly show that both methanol and aqueous extracts of *Alcea rosea* have the potential to decrease the blood glucose level. However, the decline, at 300 mg/kg b.w. of the extracts, was found to be more prominent in methanol fraction (reaching 49.2% reduction) as compared to aqueous one (24.01% reduction). It is generally believed that oxidative stress is associated with various diabetic complications like vascular atherosclerosis, retinopathy and nephropathy. Such kind of association supports our argument that antioxidants may play a vital role in the management of diabetes and its complications. In fact diabetes is marked by increased production of free radicals or impaired antioxidant defences (Bloomgarden 1997). The generation of superoxide anion radicals by glucose oxidation and its dismutation to hydrogen peroxide leads to the formation of reactive hydroxyl radicals. Continuous generation of ROS in hyperglycaemic conditions, alters the antioxidant status of various tissues (Rolo & Palmeira 2006). Keeping this in consideration, the antioxidant status of the alloxan-induced diabetic rats was also evaluated. A reduction in GR, GPx, GST, SOD, CAT (Tables 2 and 3) and GSH levels (Figure 3) was observed in untreated diabetic rats (group II) as compared to normal rats (group I). These changes may be due to the glucose oxidation and formation of reactive oxygen species. However, administration of *Alcea rosea* seed extracts significantly elevated GR, GPx, GST, SOD, CAT (Tables 2 and 3) and GSH levels (Figure 3) in liver and pancreas of diabetic rats. The ability of the extracts to restore the altered antioxidant enzymatic status in the alloxan-induced diabetic rats indicates their antioxidative potential under diabetic conditions.

Alloxan, in the presence of intracellular thiols, especially glutathione, gets reduced into dialuric acid in a cyclic redox reaction with concomitant release of reactive oxygen species like hydrogen peroxide, superoxide and hydroxyl radicals (Belkina et al. 2013; Heikkila et al. 1976; Lenzen 2008). Free radicals generated lead to lipid peroxidation which in turn releases a large amount of MDA. Quantification of MDA level in liver and pancreas should give an idea of cellular damage and apoptosis in diabetic animals (Pavana et al. 2007). In fact, MDA together with catalase and hydrogen peroxide is used as a reliable oxidative stress marker in diabetes (Packer et al. 2001). In this context, effect of different doses of aqueous and methanol extracts on liver and pancreas lipid peroxidation was studied (Figure 1). Both the extracts were found to decrease the MDA levels and as expected, methanol extract 300 mg/kg b.w. concentration showed highest anti-lipid peroxidation activity (1.63 and 1.57 nmol of MDA/g of protein in liver and pancreas, respectively) which was quite comparable to that of the standard drug metformin (1.12 and 1.40 nmol of MDA/g of protein in liver and pancreas, respectively), used in this study. Among the phytochemical constituents of medicinal plants, plant phenolics have been found to neutralize lipid peroxidation either by inhibition of chain initiation or propagation step (Rice et al. 1996). In addition to this, a significant correlation has been observed between total phenolic content of medicinal plants and their lipid peroxidation inhibitory activity (Rice et al. 1996, 1997). Furthermore, literature studies have shown that phytochemicals such as phenols, flavonoids, terpenes, and alkaloids, are also responsible for antidiabetic and antioxidant activity (Jemai et al. 2009). In order to screen out the actual candidates responsible for the results obtained in this study, phytochemical screening was carried out for *Alcea rosea*. It was observed that total phenolic content was found to be highest in methanol fraction (412.23 mg/g gallic acid equivalent) as compared to aqueous extract (149.01 mg/g gallic acid equivalent). Interestingly, it has been shown that *Alcea rosea* seeds are rich in phenolic components and are responsible for its potential antioxidant activity.

## Conclusions

Results of the present work for the first time indicate that *Alcea rosea*, in particular its methanol extract, is an active antidiabetic assay system with an ability to stimulate the cellular antioxidant status (both enzymatic and non-enzymatic) under diabetic conditions. Our findings strongly suggest that the mechanism underlying such protective effect is mediated via prevention and restoration of antioxidant status of the cell. Restoration of antioxidant currency of cell in turn normalizes the insulin secretion and thus glucose level in the blood. Future studies need to be carried out for identification and characterization of actual constituent compound(s) responsible for efficient antidiabetic activity of *A. rosea* seeds.
